# Rafting on the Evidence for Lipid Raft-like Domains as Hubs Triggering Environmental Toxicants’ Cellular Effects

**DOI:** 10.3390/molecules28186598

**Published:** 2023-09-13

**Authors:** Dorinda Marques-da-Silva, Ricardo Lagoa

**Affiliations:** 1 LSRE—Laboratory of Separation and Reaction Engineering and LCM—Laboratory of Catalysis and Materials, School of Management and Technology, Polytechnic Institute of Leiria, Morro do Lena-Alto do Vieiro, 2411-901 Leiria, Portugal; ricardo.lagoa@ipleiria.pt; 2 ALiCE—Associate Laboratory in Chemical Engineering, Faculty of Engineering, University of Porto, Rua Dr. Roberto Frias, 4200-465 Porto, Portugal; 3 School of Technology and Management, Polytechnic Institute of Leiria, Morro do Lena-Alto do Vieiro, 2411-901 Leiria, Portugal

**Keywords:** caveolae, cholesterol, endocrine disruptors, environmental toxicology, inflammatory signaling, lipophilic agents, membrane rafts, pollutants, polycyclic aromatic hydrocarbons, toxic agents

## Abstract

The plasma membrane lipid rafts are cholesterol- and sphingolipid-enriched domains that allow regularly distributed, sub-micro-sized structures englobing proteins to compartmentalize cellular processes. These membrane domains can be highly heterogeneous and dynamic, functioning as signal transduction platforms that amplify the local concentrations and signaling of individual components. Moreover, they participate in cell signaling routes that are known to be important targets of environmental toxicants affecting cell redox status and calcium homeostasis, immune regulation, and hormonal functions. In this work, the evidence that plasma membrane raft-like domains operate as hubs for toxicants’ cellular actions is discussed, and suggestions for future research are provided. Several studies address the insertion of pesticides and other organic pollutants into membranes, their accumulation in lipid rafts, or lipid rafts’ disruption by polychlorinated biphenyls (PCBs), benzo[a]pyrene (B[a]P), and even metals/metalloids. In hepatocytes, macrophages, or neurons, B[a]P, airborne particulate matter, and other toxicants caused rafts’ protein and lipid remodeling, oxidative changes, or amyloidogenesis. Different studies investigated the role of the invaginated lipid rafts present in endothelial cells in mediating the vascular inflammatory effects of PCBs. Furthermore, in vitro and in vivo data strongly implicate raft-localized NADPH oxidases, the aryl hydrocarbon receptor, caveolin-1, and protein kinases in the toxic mechanisms of occupational and environmental chemicals.

## 1. Introduction

Lipid rafts and plasma membrane domains abundant in cholesterol and sphingolipids have been reported to participate in important cellular processes, namely, in terms of the space-time dimension of signal transduction and cell signaling [1,2,3], and so act as molecular signaling hubs.

Environmental and occupational toxicants can bring about a great diversity of cellular and systemic alterations implicated in the short-term and long-term outcomes of toxic exposures [4,5,6,7,8,9]. The cellular effects of common pollutants (or their metabolites) can affect redox status, calcium homeostasis, metabolic and epigenetic programs, among other disturbances, but the key molecular targets and mechanisms of action of specific compounds remain unresolved [4,10,11,12,13,14]. 

Many environmental chemicals are lipophilic, which allows them to accumulate in the plasma membrane, but the potential role of membrane raft-like domains unleashing their cellular effects is debatable. As it will be discussed in the following sections, experimental evidence exists for the targeting of lipid rafts—on cells in vitro—by some toxicants, such as polychlorinated biphenyls (PCBs) and air pollutants [15,16,17]. In addition, environmental or occupational exposures have been demonstrated to alter the levels of raft-related lipids [18,19,20], and to disturb signaling events associated with these membrane domains [6,15,21,22,23].

Therefore, it is plausible that raft-like domains at the plasma membrane play a role as hubs/platforms for environmental and occupational toxicants to trigger or amplify their toxic actions in cells. The present work aimed to collect and discuss the existing evidence supporting this hypothesis and to identify gaps to be addressed in future research.

## 2. Lipid Rafts—Structure and Composition

### 2.1. Plasma Membrane Domains

**Figure 1:**
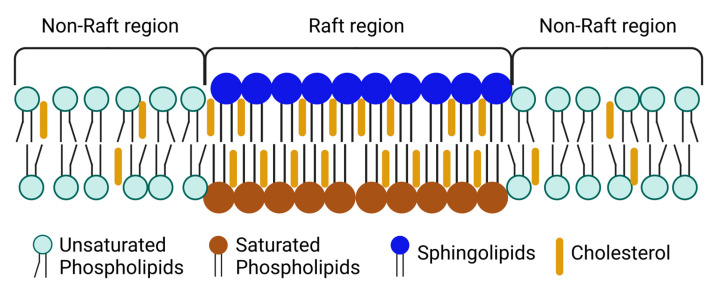
Representation of the lipidic distribution in the lipid raft and non-lipid raft regions of the cell membrane. Sphingolipids are enriched in the outer leaflet of the raft region, along with saturated phospholipids present in the inner leaflet. Lipid rafts present a more packed organization than non-raft regions. Figure created with BioRender.com.

The lipid rafts concept marked a change in the understanding of the plasma membrane organization and function. Before it, the plasma membrane was understood by Singer and Nicholson’s classical membrane fluid mosaic model. Nevertheless, the new concept amplified the understanding related to the structure and function of the plasma membrane [24,25] into a more complex structure where lateral diffusion of membrane domains is possible. Lipid rafts are described as plasma membrane compartments highly enriched in cholesterol, sphingolipids, and phospholipids containing saturated fatty acids (Figure 1) that support different functions of the cell, such as signal transduction [24,26,27]. By norm, the lipid rafts are defined as membrane subdomains enriched in cholesterol and sphingolipids containing saturated acyl chains as represented in Figure 1 [1,24]. The idea of rafts derives from the floating property promoted by the sphingolipids’ enrichment in the outer leaflet of the membrane (Figure 1) [28].

The frontier between floating membrane domains and larger and more stable membrane subdomains is not clear. Lipid rafts can vary from highly transient domains to larger and more stable structures [29]. In this sense, the extent of lipid raft enlargement depends on the organism, cell type, and composition of the raft [30]. In eukaryotic cells, lateral compartmentalization of the plasma membrane into microdomains has been described, and yeast cells present very well-defined membrane compartments [30]. The membrane compartmentalization into subdomains is crucial for the physiological functions of the cell [31], in accordance, the structure of these domains is distinct depending on the cell specialization [32,33,34]. Nevertheless, the compartmentalization mechanisms are complex and still need to be better understood, with a variety of possibilities for membrane subdomains to co-exist, such as stable domains, transient compartments, or nanodomains [31].

In many mammalian cells, caveolae are the most abundant membrane subdomains [35]. They were first reported in 1955 [36] and are also considered a sub-type of lipid raft in the form of small flask-shaped invaginations due to the presence of caveolin [37,38,39]. Although lipid rafts have similar components and comparable functions, they are more diverse in terms of size and considered more dynamic than caveolae [40]. Caveolae, on the contrary, are considered stable structures in the plasma membrane [41].

The physical-chemical characteristics of caveolae facilitate their identification by electron microscopy, while the existence of lipid rafts in living cells remains to be clearly demonstrated [40]. The direct observation of lipid rafts that would clarify the existence of these structures in living organisms is challenging due to their nanoscale dimensions and limited lifetime. For this reason, some authors are critical of the concept of lipid rafts [42,43] and the methods used to study these domains are discussed [44]. Yet, scientific advances are being made in this field [45] and a collection of fluorescent lipid probes that preferentially partition into raft and non-raft domains has been suggested recently [46]. 

Despite the doubts of the scientific community about the appropriate nomenclature of membrane raft-like domains and methodological limitations to proof the existence of lipid rafts, from the perspective of Sevcsik and Schütz (2015), the concept of lipid rafts is defended until methodological evidence is clarified [42]. Having this discussion in mind and the common properties shared by the different membrane raft-like domains, the reader will be guided throughout this review on the use of the general term “lipid rafts” as an umbrella for the different nomenclatures that include rafts, detergent-resistant membranes (DRMs), and caveolae [1,42].

### 2.2. Properties of Lipid Rafts and Composition

The lipid rafts, which originate from the interactions between sterols and saturated lipids, form a liquid-ordered phase that is tightly packed, distinct from the typical disordered lipid phase regarding flexibility and permeability [24]. Regen (2020) presented lipid rafts with a central role for cholesterol in promoting favorable or repelling interactions depending on the lipid with which cholesterol interacts, i.e., high-melting lipids or low-melting lipids, respectively [47]. Moreover, the lipid composition of these rafts was described as being different between the exoplasmic and cytoplasmic leaflet faces. Cholesterol and sphingolipids are described as being present in the outer face, while cholesterol and phospholipids are in the inner leaflet of these microdomains (Figure 1) [48]. The difference in lipid composition is justified by the difference in lipids’ function, with the phospholipids forming the lipid bilayer and the sphingolipids modulating fundamental cell processes such as apoptosis [49]. Furthermore, there is a correlation between the complexity of membrane composition, properties, and functions. The presence of sphingolipids and sterols in eukaryotic cell membranes makes vesicular trafficking possible, impacting the establishment and maintenance of distinct organelles [50].

In general, cholesterol is an essential player in the different types of lipid rafts. The deprivation of cholesterol reduced the number of caveolae in the cell, showing its essential role in the formation of these structures [51], but its relevance doesn’t stop here. Its key role in the cell requires its synthesis, homeostasis, and efflux to be maintained through a free cholesterol concentration gradient between the endoplasmic reticulum and the plasma membrane that is preserved by a tight feedback control mechanism [52]. Moreover, intracellular cholesterol trafficking regulates membrane rafts and consequent cell signaling [52,53]. To investigate the effect of cholesterol in lipid rafts’ cellular mechanisms, different chemical agents are used as methodological approaches to experimentally remove cholesterol. One example of these cholesterol-depleting agents is nystatin, as described in [54], but methyl-ẞ-cyclodextrin is probably one of the most commonly used to test the presence or function of a protein in the cell microdomains [55,56,57,58]. 

A well-described lipid specific to membrane rafts is the GM1 glycosphingolipids or ganglioside [59,60], which is highly relevant for the function of diverse signalosomes [61]. The GM1 glycosphingolipids are cellular receptors for the cholera toxin B-subunit (CTxB) [62], a protein widely investigated and associated with lipid rafts [63]. This relationship makes CTxB a lipid raft marker extensively used in studies of membrane biology and biophysics [19,64]. In the laboratory led by Professor Carlos Gutierrez-Merino, CTxB was useful to demonstrate the clustering of plasma membrane-bound cytochrome b5 reductase within "lipid raft" microdomains in neurons in vitro and the cerebellum cortex of adult rats [65,66]. In more recent works developed by other groups, the presence of GM1 gangliosides near specific neuronal proteins is starting to be revealed as an interfering factor for their function [67,68].

The lipid rafts are also composed of membrane proteins that can be recruited by different molecular mechanisms, such as post-translational modifications like palmitoylation or protein-protein/lipid interactions, among others [69]. Moreover, the cooperation between lipids and proteins is essential for membrane organization. For example, some proteins, like cytoskeletal components, can regulate lipid domains, or, on the other hand, protein oligomerization can contribute to the clustering and stabilization of raft domains, as reviewed by [43]. As described before, a specific type of raft is known as caveolae (“little caves”), containing caveolin proteins and forming the innermost layer of the caveolar coat [39,70]. Caveolae form invaginations enriched in cholesterol, sphingolipids, and lipid-modified proteins such as H-Ras, with the caveolins being suggested to act as concentration and organization agents of these domains [71]. Other proteins were also reported to be enriched on lipid rafts, and in the specific case of flotillin, it was described as stabilizing caveolin-1 [71,72]. For better comprehension, in 2003, a study identified three different types of proteins: raft proteins, raft-associated proteins, and nonspecific proteins [73]. Proteins like flotillins and caveolin-1 were identified as raft and raft-associated proteins [73]. Moreover, another work reviewed the properties and functions of permanent raft-resident proteins and temporary raft-resident proteins [69]. Again, the structural proteins caveolins and flotillins were identified as permanent raft-resident proteins, while other proteins like TNF receptor 1 and NADPH oxidase were identified as temporary raft-resident proteins [69]. 

At the experimental level, having proteins identified by different studies as characteristic of lipid rafts allows us to use them as lipid raft markers in DRMs. DRMs are considered representative of membrane lipid rafts [1] since their obtention takes advantage of the lipid rafts’ insolubility in non-ionic detergents at 4 °C and the consequent use of sucrose-density gradients [74]. This methodological approach was used in the laboratory of Professor Carlos Gutiérrez-Merino to investigate the presence of functional proteins in the membrane microdomains of cultured cerebellar granule neurons [58,75,76,77,78]. But fluorescence methods, such as Foster resonance energy transfer (FRET) and high-resolution microscopic techniques, are useful when studying lipid rafts [45,46,52]. These tools allow for carrying out studies related to signal transduction and cellular responses associated with lipid rafts, which can be considered a technical challenge due to the nanoscale scale of these cellular structures, estimated to be 5–80 nm [52].

### 2.3. Lipid Rafts as Platforms for Signal Transduction in Cells

As described in Section 2.1, despite the immensity of works on lipid rafts, their existence, nature, and function in vivo are still an open question for some authors [43], due to their dynamic nature and small occurrence scale [52]. Although their relevance for cell function has been proposed since the first reports of lipid rafts [27], there is accumulating evidence for their existence and role in cell biology [52]. 

It is expected that the presence of proteins in lipid rafts in nanoscale dimensions favors the ability of lipid raft components to respond to diverse stimuli [52]. For example, proteins such as calcium channels, transporters, and redox proteins were identified in lipid raft microdomains of cerebellar granule cells [75,76,77,78,79]. These nanodomains were suggested to act as “calcium micro-chip-like structures,” forming a focalized redox/calcium integrative structure for fast and efficient cross-talk between calcium and redox signaling in neurons [77], which can be highly relevant to avoid distortion of cytoskeleton-linked lipid raft structures more prone to oxidation such as actin [80]. Additionally, the removal of cholesterol impacted cytosolic calcium homeostasis, leading cells to a pro-apoptotic state, showing that the proximity between the investigated proteins guarantees their function in cell signaling [58]. Indeed, the consequences of removing cholesterol and having non-functional proteins because they are dissociated from lipid rafts have long been described [48]. These physiological advantages are representative of the cellular benefit of membrane compartmentalization generated by the lipid rafts, but other works widely reflect the effect of lipid rafts in cell signaling [1,81]. And, new cellular mechanisms associated with raft membranes are being continuously described, such as inflammation [53], tyrosine kinase receptor, T cell antigen receptor, and estrogenic signaling [82,83,84], as well as signaling associated with cancer [3,85,86]. In the next section, a detailed description is provided for the cellular mechanisms associated with lipid rafts.

## 3. Lipid Rafts in Cell Signaling and Disease

Cell maintenance and proper functioning rely on the different intra and extra-cellular processes, their flux, equilibrium, and control. And lipid raft-like domains are described as contributing to different cellular processes as described previously [1,53,81,82,83,84].

### 3.1. Calcium and Redox Signaling in Neurodegeneration

Different studies investigated the importance of lipid rafts in neurodegeneration, some pointing to neuroprotective effects while others pointed to neuropathological consequences. In this sense, different points of view are reflected in [87] and a reconfiguration of membrane rafts was proposed as a strategy to counteract mechanisms associated with Alzheimer’s disease (AD), Parkinson’s disease, and amyotrophic lateral sclerosis [88]. For example, effects on the aggregation of amyloid and on the processing of the amyloid precursor protein associated with lipid rafts have been reported [17,89]. Nevertheless, how chemical toxicants can affect these mechanisms is not yet known. Recently, DTT was shown to provoke loss of the postsynaptic density protein 95, a protein reported in complexes of raft and postsynaptic proteins [90] and this effect was related to altered levels of the amyloid precursor protein [91].

In terms of understanding the disrupting effects of oxidative and nitrosative stress in lipid rafts, they are well described in neuroimmune disorders [92]. In the neuronal cell line N27, the disruption of lipid rafts blocked androgen-induced oxidative stress in cells by decreasing the localization of the membrane androgen receptor in lipid rafts [54]. In this case, as in another study with brain endothelial cells [16], the production of reactive oxygen species (ROS) was ascribed to raft-localized NADPH oxidase, a superoxide-producing enzyme. On the contrary, lipid rafts in cerebellar granule neurons were described as ROS generation points since they cluster the cytochrome b5 reductase, leading to exaggerated plasma membrane-focalized superoxide anion production and oxidative stress-mediated apoptosis [66,78]. This same protein was found close to lipid raft regions in adult rat cerebellum neurons [65]. Additionally, in primary fibroblasts from familial AD patients, the amyloid beta oligomers were recruited to membrane rafts leading to lipid peroxidation and deregulation of calcium homeostasis [93]. A similar effect was observed in cerebellar granule neurons, where the cytosolic calcium homeostasis was affected by the binding of amyloid-calmodulin complexes to lipid rafts [94]. Indeed, calcium deregulation is implicated in neurodegeneration [95,96,97] and, in different neurons, the removal of cholesterol affected the calcium homeostasis controlled by different calcium proteins, leading, in the case of cerebellar granule neurons, to apoptosis [58,98,99]. This interplay between oxidative stress and calcium homeostasis in neurons related to lipid rafts is described by different authors [77,99]. Thus, if we consider that the amyloid precursor protein and amyloid peptides occur in the membrane rafts that are implicated in nitrosative processes and modulation of calcium signaling in different neurons [17,89,94], the relevance of these structures—allowing the interplay between both signaling actors—in neurodegeneration and other pathologies deserves further investigation.

### 3.2. Inflammation and Atherosclerosis

Another example of lipid rafts’ relevance in cell signaling is their implication in TNFα-mediated signaling. A study showed that cholesterol sequestration from lipid rafts inhibits the activation of the nuclear factor kappa-light chain-enhancer of activated B cells (NF-κB) pathway and therefore induces the switch of TNFα-mediated responses toward apoptosis [100]. Moreover, the effects on TRL4 signaling in macrophages are also influenced by lipid rafts. There are different mechanisms by which lipid raft perturbations—including intracellular and extracellular cholesterol trafficking—regulate the innate immune response [53]. Recently, in the context of neuroinflammation and pain processing, the concept of inflammaraft was proposed to represent the lipid raft platforms that initiate an inflammatory response [101]. These membrane microdomains function as a framework for inflammatory signaling, containing receptors and signaling molecules such as TRL4, ion channels, and enzymes [101]. 

Exposure to ambient and diesel exhaust particulate matter (PM) and heavy metals like lead and mercury induces inflammation and endothelial/vascular dysfunction, which is associated with altered vasoconstriction/vasorelaxation and the development of atherosclerosis and cardiovascular diseases [5,12,14]. Noteworthy, proteins controlling Ca^2+^ signaling and the endothelial nitric oxide synthase (eNOS or NOS3) are localized in caveolae of endothelial cells and play central roles in regulating blood pressure and flow, angiogenesis, and vascular inflammation [14,16,102,103]. In these cells, when cytosolic or local microdomain calcium levels rise, calcium-calmodulin activates NOS3 and nitric oxide production by displacing the enzyme from caveolin-1 [14,103]. At the disease level, a review explored the relevance of the plasma membrane microdomains in the inflammation associated with atherosclerosis [104]. Moreover, in vivo evidence highlights the significance of lipid rafts [56,57] and inflammarafts were observed in nonfoamy macrophages in atherosclerotic lesions [105].

### 3.3. Immune Regulation

The plasma membrane microdomains play an important role in immune regulation. One example is the requirement of lipid rafts for target internalization by the platelet IgG Fc receptor (FcγRIIa). Although the receptor activities are independent of the receptor localization [106]. 

In T and B cell lines, the lipid rafts are also required for the recruitment of different components of the death-inducing signaling complex (DISC), allowing efficient Fas signaling and apoptosis [107]. Also in T cells, the lipid rafts were shown to be implicated in the induction of apoptosis through the clustering of DISC protein components [108]. Moreover, the disruption of lipid rafts suppressed the drug-induced DISC assembly and apoptosis [108]. Regarding the effects of disrupting lipid rafts in T cells, it was shown that it affected T cell receptor (TCR) signaling [83] and receptor nanoclusters could be involved in enhanced memory sensitivity compared with naive T cells [109]. Indeed, a study revealed that T cell responses to TCR stimulation will depend on the level of lipid ordering in the plasma membrane. The authors observed that high membrane order promoted T cell proliferation, while low membrane order derives from insensitive T cells [110].

The deregulation of T and B cell signaling can conduct to autoimmune diseases. Diverse studies point to the relevance of the lipid raft signaling platform in unbridled T cell responses in systemic lupus erythematosus (SLE). The T cells of SLE patients showed increased expression of the raft-associated GM1 [111] and different lipid raft compositions were associated with higher and frontloaded calcium responses [112]. Moreover, B lymphocytes of SLE patients showed altered expression of kinases and phosphatases and altered interaction with lipid rafts or translocation into these microdomains [113]. 

In the case of autoimmune rheumatic disease, a study investigated ex vivo primary human CD4^+^ T cells, observing that the response to TCR stimulation depends on the ordering of the lipid membrane. This study identified that the patients’ T cells showed a distinct membrane order when compared with the T cells of healthy volunteers [110]. And a recent study highlights the relevance of cholesterol-dependent membrane order in CD4^+^ T cell signaling [114].

Another interesting example of the rafts’ involvement in disease is the study case of raftlin, an abundant protein in the lipid rafts of B cells and long known as essential for the functioning of these lipid rafts and the associated B-cell antigen receptor signaling [115]. The fact that the development of chronic rhinosinusitis with nasal polyps is related to the deficiency of raftlin in the nasal polyp tissue [116] points to the critical role of lipid raft integrity in this tissue and disease.

### 3.4. Hormone Signaling

The thyrotropin receptor’s (TSHR) functions are regulated by lipid rafts [117]. And these receptors are key regulators for thyroid growth and function, and interaction with G proteins results in different cellular responses such as hormone synthesis and secretion but also cell proliferation or survival. In a cell model, thyrotropin increased the TSHR localization in plasma membrane rafts, apparently necessary for the subsequent internalization of the receptor [19]. Moreover, the same mechanism of lipid raft-mediated hormone signaling is described for the luteinizing hormone receptors. In this sense, the translocation of the hormone-occupied luteinizing hormone receptors into lipid rafts was reported as an optimizing condition for signaling [118]. 

The receptor tyrosine kinases, which also play a relevant role as hormone receptors, are associated with different cellular processes after ligand binding. It should be recalled that several environmental toxicants behave as endocrine disruptors, and some effects may involve G protein-coupled receptors [4,119] and/or signal-transducing protein kinases associated with lipid rafts—Src, MAPKs, JAK2, and LRRK2 (as detailed in Section 4). The clustering of receptors and related kinases into the different lipid raft types along with the affected signaling pathways is widely described, and a review is recommended for further details [82]. In the same way, the nongenomic effects of estrogens were also described as being related to the presence of estrogen receptor (ER) subpopulations in lipid rafts [84]. The activation of these plasma membrane ER triggers rapid cell responses, while the classic effects mediated by intracellular ER take a few hours [84].

The interaction of estrogen with the ER located in signalosomes mainly present in lipid rafts is described as conducting preventive mechanisms counteracting AD [120]. Indeed, the malfunction of ER-signalosomes was also discussed in menopause conditions [121]. More recently, the same group showed a slight increase in six proteins associated with the ER-signalosome—including ERalpha, caveolin-1, and flotillin—in the preclinical stages of AD [122]. In addition to neurons, ERalfa or ERbeta have been found at caveola/lipid rafts or associated with raft proteins in endothelial cells, vascular smooth muscle cells, cardiomyocytes, platelets, lymphocytes, and cancer cells [84].

The ERalfa at membrane caveolae associates with G proteins, NOS3, Src kinases, phosphoinositide 3-kinase (PI3K)/Akt, JAK/STAT, and MAPKs [84]. This cluster of proteins partially coincides with the rafts-associated protein network further described in Section 4 and can underlie changes in cytosolic calcium and cAMP levels, dysregulation of nitric oxide production, and MAPK, PI3K, JAK/STAT, or Src/STAT pathway activation, processes implicated in environmental toxicants’ exposure, inflammation, and carcinogenesis [4,5,11,12,123,124]. These ER signalosome complexes could allow estrogen disruptors targeting lipid rafts to modify hormone signaling without direct interference with receptor-hormone binding.

A membrane androgen receptor (AR45) has also been found in the plasma membrane rafts of neuronal, prostate, and Sertoli cells [54]. In N27 neurons, AR45 is associated with caveolin-1 and NADPH oxidase, and under oxidative conditions, testosterone amplified cell stress and activated caspase-3 in a cholesterol-dependent way. Furthermore, the disruption of lipid rafts was reported to decrease membrane androgen receptors and their internalization [54]. 

### 3.5. Cell Communication

Also in cell communication processes, the lipid rafts reveal themselves to be essential either by favoring exosome uptake or biogenesis [125,126] or by influencing the co-localization of connexin-43 with caveolin-1 [127]. 

Indeed, connexin-43 allows direct cell-cell communication through participation in gap junctions and is also suggested to take part in cellular fine-tuned regulation mechanisms like cell cycle regulation [128] which highlights the role of lipid rafts in these mechanisms. Moreover, the spatiotemporal dimensions are becoming increasingly relevant to understanding membrane trafficking mechanisms, and lipid rafts are one of the players in the automation of these biological processes [2]. 

### 3.6. Cell Death and Cancer

Additionally, and complementary to the role of cholesterol described earlier, the depletion of this sterol can also affect apoptosis and proliferation mechanisms since it activates a protein responsible for the maintenance of cellular pH, the Na+/H+ exchanger 1 (NHE-1). In this case, cholesterol depletion is associated with a relocation of the protein outside the microdomains, leading to its activation [129]. But from the therapeutic perspective of cell signaling, cholesterol was shown to play a critical role in the resistance of glioblastoma cells to temozolomide. The cell viability of non-resistant U251 cells increased by decreasing intracellular cholesterol, whereas the addition of cholesterol decreased cell viability [130,131]. This effect occurred via the accumulation and activation of a protein of the tumor necrosis factor receptor family—death receptor 5—in the lipid rafts that affect cell death mechanisms via caspase signaling [131]. 

Other apoptotic and anti-apoptotic signaling pathways were also described as being triggered by lipid rafts [132]. The apoptotic signaling dependent on lipid rafts can derive from the receptors and channels embedded in the plasma membrane, such as Fas, CD5, CD20, and Trpc-1, or via protein kinase proteins like Akt (or PKB), JNK, Src kinases, and protein kinase C (PKC) family proteins [132]. Lipid rafts are implicated in the operation of different signaling mechanisms regulating cancer cell survival, death, invasion, and metastasis [85,86]. It is especially impressive that lipid rafts were revealed to be essential to promoting or inhibiting the different cell signaling pathways associated with distinct stages of metastasis, such as angiogenesis, epithelial-to-mesenchymal transition, migration, transendothelial migration, cell death, and adhesion [3].

## 4. Effects of Environmental Toxicants in Lipid Rafts Organization and Signaling

Considering the high relevance of lipid rafts in cell signaling and disease, how environmental toxicants affect such cellular mechanisms deserves to be explored.

If we look at the Gene Ontology (GO) database, 109 proteins are listed in the “Plasma membrane raft” class of Cellular Component (GO:0044853). And, as expected, these proteins are associated with a great diversity of cellular and molecular functions, including responses to chemical stimuli and cellular stress.

**Figure 2:**
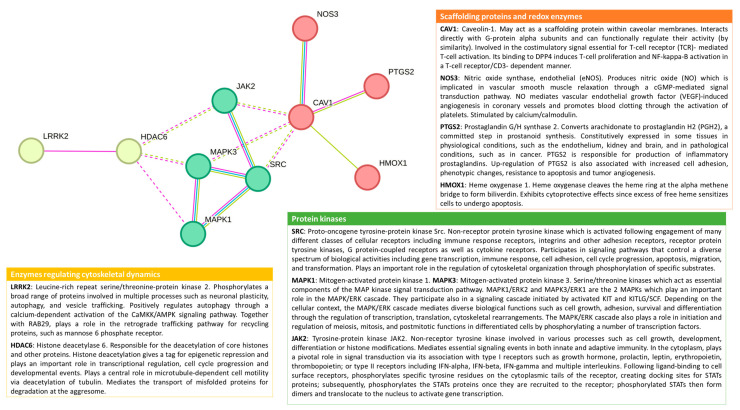
Interaction network of the proteins closely related to membrane lipid rafts and involved in cellular responses to chemical stress. The network was generated with STRING https://string-db.org (accessed on 15 April 2023) entering proteins classified simultaneously in Gene Ontology (GO) “Plasma membrane raft” cellular component (GO:0044853) and Biological Process “Cellular response to chemical stress” (GO:0062197). Physical subnetwork (edges indicate physical association) of proteins with a minimum Interaction score of 0.400. Different colors represent the three clusters of proteins obtained by both K-means and MCL clustering methods. The protein descriptions were adapted from UniProtKB https://www.uniprot.org (accessed on 15 April 2023).

**Figure 3:**
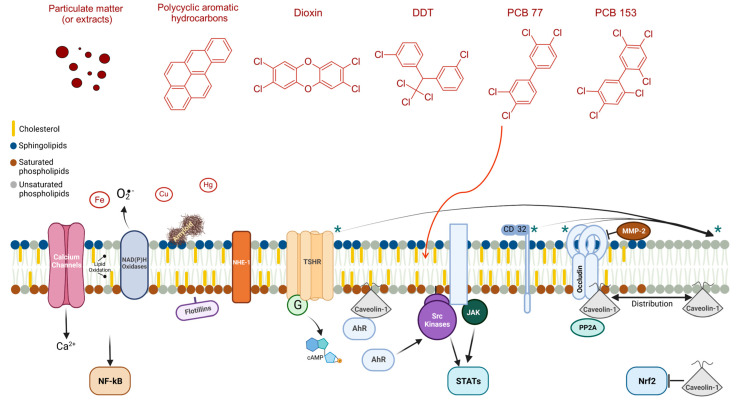
Membrane lipid rafts components and associated signaling pathways implicated in the cellular effects of environmental toxicants. The proteins marked with an asterisk (*) are distributed between raft and non-raft domains, and environmental toxicants were described to displace them from lipid rafts. Abbreviations: AhR, Aryl hydrocarbon receptor; cAMP, Cyclic adenosine monophosphate; DDT, 1,1,1-trichloro-2,2-bis(p-chlorophenyl)-ethane; G, G proteins; JAK, Janus kinase; MMP2, Matrix metalloproteinase-2; NF-kB, factor nuclear kappa B; NHE-1, Na+/H+ exchanger 1; Nrf2, Nuclear factor (erythroid-derived 2)-like 2; PCB, Polychlorinated biphenyl; PP2A, Protein phosphatase 2A; Src, Proto-oncogene tyrosine-protein kinase; STATs, signal transducer and activator of transcription; TSHR, thyrotropin receptor. Figure created with BioRender.com.

Under the scope of this work, by refining the list of proteins to those classified in the biological process “Cellular response to chemical stress” (GO:0062197), the protein association network shown in Figure 2 could be obtained. The more enriched KEGG pathway is the vascular endothelial growth factor (VEGF) signaling pathway—proteins Src, MAPK1, and 3 (also known as ERK2 and 1, respectively), PTGS2, and NOS3—a pathway tightly connected to angiogenesis, the permeability of endothelial cells, and tumor growth. Also to be noted, the network contains several protein kinases, components of the mitogen-activated protein kinase (MAPK), and other important signal transduction pathways controlling a variety of biological processes, including cytoskeletal arrangements, regulation of cell fate, and immune response. Hence, the proteins identified in this network seem to be probable mediators of cellular responses to toxic chemicals targeting plasma membrane rafts. Indeed, as discussed in the next sections, some of these proteins were described as involved in the cellular effects of environmental toxicants. But other proteins, the lipids present in lipid raft-like domains, and the lipid rafts themselves are also described as targets of environmental toxicants. At the end of this section, Figure 3 compiles a schematic overview of the reported evidence found in the literature and distributed along the next subsections.

### 4.1. Accumulation of Environmental Toxicants in Lipid Rafts and Associated Cellular Effects

Being the cell membrane a barrier between extracellular and intracellular space, it is expected that lipophilic organic compounds accumulate in this lipidic environment (Figure 3). A study showed that lipophilic hydrocarbons accumulate in the lipid membrane, affecting the structure and properties of the membrane [133]. In liposomes, the pesticides 1,1,1-trichloro-2,2-bis(p-chlorophenyl)-ethane (DDT) and lindane were found to intercalate between the fatty acyl chains of membrane phospholipids [134,135]. Moreover, another study investigating 240 organic compounds showed that partition coefficients for membrane lipids are higher when compared to the partition coefficient for storage lipids [136]. And specifically, in the case of PAHs, it was shown that depending on the compound investigated, it could mix or not with phospholipid monolayers. B[a]P, among others, was shown to incorporate into the membrane [137]. But other cases of toxic accumulation in the plasma membrane were described, such as the case of 2,4,6-trinitrotoluene (TNT) and its metabolites [138]. Toxicant metabolites, even if more hydrophilic than the parent molecules, can be incorporated into lipid membranes and modify lipid-lipid and lipid-protein interactions. For instance, 1-hydroxypyrene, the main metabolite of pyrene and a biomarker of PAH exposure, occupies the water-inaccessible interior of liposomes, apparently residing in a quite rigid environment [139].

Studies with clear evidence for the accumulation of environmental toxicants on the lipid raft domains of cell membranes are scarce. The enrichment of lipid rafts on toxic species can occur by binding to proteins, as proposed for copper ion complexation by amyloid in neuronal lipid rafts [89]. Another study demonstrated the accumulation of PCB77 in the caveolae-rich fraction [102] and further studies revealed that exposure to PCB77 strongly impacts vascular inflammation mechanisms [102,140]. Interestingly, in both studies, caveolin-1 silencing attenuates the effects provoked by PCB77 either in vitro or in vivo [102,140]. There was an increase in MCP-1 cytokine levels in endothelial cells exposed to PCB77 [140]. Depicting the molecular mechanism implicated in the increase of MCP-1 levels produced by PCB77, different pathways such as the aryl hydrocarbon receptor (AhR), p38, and JNK signaling were identified [140]. Moreover, PCB77 provoked an increase in caveolin-1 and CYP1A1 levels in the same cells, showing that PCB77 promoted the binding of the AhR to caveolin-1 [102]. 

AhR is a transcription factor activated by several major environmental toxicants, such as dioxins, some PAHs, pesticides, and PCBs, regulating the transcription of CYP450 enzymes (e.g., CYP1A1) and matrix metalloproteinases (MMP), among other genes [141]. However, the relevance of AhR non-genomic functions has been discussed, namely the modulation of cytosolic calcium and caveolin-1 distribution [4,14,142]. In line with the data above on endothelial cells, a subpopulation of AhR could be found in caveolin-1 DRMs from fibroblasts and hepatoma cells, and the two proteins could immunoprecipitate each other [142]. Moreover, AhR expression promoted the localization of caveolin-1 at fibroblasts’ DRMs, playing an important role in controlling cell adhesion and migration. Mechanistically, a non-canonical AhR signaling pathway has been proposed to activate c-Src, at least in response to dioxin (Figure 3), and c-Src activity participates in the regulation of caveolin-1 expression and phosphorylation [14,141,142].

Oxidative stress was also associated with PCB77 exposure [102,140]. The aggregation of PCBs into lipid rafts and induction of oxidative stress has been previously suggested, as well as antioxidant compounds that could protect against the damaging effects of PCBs [143,144]. PCB-induced oxidative stress can be related to the inhibition of nuclear factor (erythroid-derived 2)-like 2 (Nrf2) pathways, which are important for the induction of antioxidant genes like heme oxygenase-1 (HMOX1). Caveolin-1 silencing increased Nrf2 transcriptional activity in PCB126-incubated cells, and endothelial cells from caveolin-1^−/−^ mice treated with PCB126 showed higher expression of antioxidant genes [145].

The same group observed that disruption of caveolae attenuated the B[a]P-induced expression of the intercellular adhesion molecule-1 (ICAM-1), again revealing a pro-inflammatory mechanism involving AhR signaling dependent on caveolae [146]. Thus far, the accumulation of B[a]P in lipid rafts is not clearly evident. Therefore, this type of evidence regarding the accumulation of environmental toxicants in lipid rafts would amplify the perception of the relevance of these lipid structures in environmental toxicology.

### 4.2. Alterations in Rafts’ Lipid Composition and Associated Cellular Effects

#### 4.2.1. Disruption of Lipid Rafts

Alteration of lipid raft properties and composition by environmental toxicants has been reported in different studies [18,19,55,147].

A concrete effect on lipid rafts was described for iron associated with silica exposure in macrophages, where disruption of lipid rafts was observed together with an increase in superoxide, lipid oxidation, and cytokine production [55]. Iron chelators inhibited all the effects, and an inhibitor of phospholipase C (PLC) blocked cytokine production. The authors proposed that in the presence of a low noncytotoxic concentration of silica, iron prompted lipid oxidation, disrupting lipid rafts, and signaling to produce cytokines by way of PLC and NF-kB. The NF-kB is an essential regulator of inflammation and innate immunity, controlling the transcription of a variety of effectors including cytokines, ICAM-1, and cyclooxygenase-2 (coded by the PTGS2 gene, Figure 2).

Lipid oxidation and oxidative stress were also observed in lipid raft fractions from both the cerebral cortex of mice and neuronal cell models of AD exposed to airborne PM with pro-amyloidogenic activity [17]. Pointing to the critical role of the oxidative modifications, the PM triggered rapid increases in nitric oxide and hydroxynonenal in the cells, and the antioxidant N-acetyl-cysteine attenuated the PM-induced increase in lipid raft amyloidogenesis [17].

The effect of B[a]P in membrane remodeling was investigated by using rat liver F258 epithelial cells, revealing modification of lipid rafts [18]. Previously, the authors described that B[a]P affected membrane fluidity, inducing apoptosis [148] and a deeper research design showed that exposure to this toxicant changed the cellular distribution of the ganglioside-GM1, altered fatty acid composition, and decreased the cholesterol content of lipid rafts in different cell types [18,149]. This effect could be explained by the reduced expression of 3-hydroxy-3-methylglutaryl-CoA reductase (HMG-CoA reductase), observed with as low as 50 nM of B[a]P [18], since HMG-CoA reductase catalyzes the rate-limiting step of cholesterol synthesis [150]. Moreover, the authors went further, and the experimental results obtained suggest that AhR and CYP1 pathways are implicated in the membrane remodeling triggered by B[a]P [18]. Indeed, the toxicity of this compound is closely linked to the activation of the AhR and metabolization by CYP450 enzymes [4]. Furthermore, the membrane remodeling by B[a]P was considered an apoptosis-inducing intracellular alkalinization event [18] that was then shown to be related to the relocation of NHE-1 from lipid rafts promoted by B[a]P [151]. This alkalinization event was prevented by docosahexaenoic acid (DHA) and eicosapentaenoic acid (EPA), which showed to act as protector agents precisely by affecting B[a]P effects at the plasma membrane and NHE-1 activity [152]. 

B[a]P was also suggested as a destabilizing agent of lipid raft structure [147]. By using rat hepatocytes, 100 nM of B[a]P provoked diffuse staining of the ganglioside-GM1 compared to a no-treatment situation, but supplying cells with cholesterol counteracted the B[a]P effect. Moreover, the addition of cholesterol also inhibited further cellular effects of B[a]P such as decreased cell viability and ATP content. Additionally, B[a]P provoked lipid oxidation that was then reversed by a lipid raft disrupter [147]. Furthermore, the authors observed that B[a]P effects were aggravated by further exposing the cells to ethanol and that both toxicants triggered the permeabilization of lysosomes through the plasma membrane [147]. The effect of B[a]P exposure on the decrease of membrane cholesterol was also reported in human macrophages, where lipid raft stability is affected along with their activation mechanism [15]. Finally, the effect of B[a]P on membrane dynamics and lipid components was recently investigated in vivo [22]. In the lung tissue of B[a]P-exposed animals, it was observed a decrease in the levels of cholesterol, glycolipids, microviscosity, and anisotropy, together with an increase in total lipids and phospholipids [22]. Additionally, alterations in membrane fluidity, polarization, and order of membrane were also described in lung tissue [22]. 

#### 4.2.2. Alterations in Membrane Lipids

Together with disruption, changes in the membrane order and levels of raft-related lipids are also reported after exposures to other organic and inorganic pollutants. By incorporating into the bilayer, DTT decreased the phase transition temperature of model membranes [134,135] and depleted rafts of their phosphoglycolipid and cholesterol contents [19]. In Wistar rats, it was observed that aluminum treatment caused disorganization of the lipid bilayer in erythrocytes [153]. PCB52 is described in two different studies for its effect on cell membrane fluidity, either by increasing it in mouse thymocytes [154], or by decreasing it in cerebellar granule cells [155]. Moreover, exposure of DU145 cells to 1 microM of the pesticide aldrin led to a decrease in phospholipids and sphingolipids, whereas aroclor 1254 (PCB 82) and chlorpyrifos caused an increase in certain phospholipids and glycosphingolipids in the chloroform and methanol extracts [156]. On a different front, exposure to the anti-cancer cardiotoxic drug doxorubicin downregulated the HMG-CoA reductase in cancer cells, as previously reported for B[a]P [18], with the consequent reduction of cholesterol and lipid rafts [157]. These actions of doxorubicin were closely connected to the inactivation of the epidermal growth factor receptor (EGFR)/Src signaling pathway and activation of caspase-3, effects that were ameliorated by cholesterol supplementation. Interestingly, other work observed doxorubicin-induced caspase-3 activation in association with lipid oxidation and NADPH oxidase activation [158], similar to the reported for B[a]P and PM organic mixtures [4,147,159]. These environmental toxicants and others are ligands of AhR that, in turn, can activate membrane NADPH oxidase [4,141].

The membrane raft-associated AhR discussed before and their possible functions as signaling mediators, in addition to being transcription factors, might play important roles in fibroblasts, hepatocytes, and endothelial cells’ responses to dioxin-like pollutants, especially under conditions of membrane cholesterol deficit [14,142]. A dichloromethane extract of diesel exhaust particles, containing the PAHs phenanthrene, fluoranthene, pyrene, and chrysene, among other organic compounds, was found to disorder the plasma membrane of human microvascular endothelial cells, an effect that was prevented with cholesterol supplementation of the media [14]. The same extract and others with more lipophilic compounds triggered a rapid increase in intracellular calcium, which was substantially reduced when AhR was pharmacologically inhibited or knocked down via siRNA. However, the fast kinetics of this response are inconsistent with a mechanism of transcriptional regulation and can only be explained by a nongenomic AhR signaling pathway. Functional interaction of the rafts-associated AhR with caveolin-1 and Src signaling [14,141,142] indicates that caveolin-1, cytosolic calcium, NADPH oxidases, and Src kinases can transduce the destabilization of cholesterol-rich plasma membrane domains by organic toxicants (Figure 3). In neurons, the strongly hydrophobic pesticide rotenone also induced a fast cytosolic calcium increase, but by way of specific calcium channels expressed in these cells at membrane rafts [13,77]. 

#### 4.2.3. Alterations in the Levels of Raft-Related Lipids

In humans, it is well established that cadmium, lead, and mercury exposures increase serum cholesterol levels [12]. Correlations were also detected between blood concentrations of arsenic and mercury, and especially manganese and zinc, with alterations in the levels of plasmenyl phospholipids and sphingomyelin [160]. A metabolomic study of mice exposed to PM also uncovered alterations in the blood levels of glycerol (phospho)lipids, sphingolipids, and lysophospholipids and in pathways involved in the metabolism of fatty acids and sterols, among other critical metabolites and hormones [20].

However, how these changes in lipid levels reflect in cell membrane lipid rafts has yet to be investigated. In one study, exposure to manganese induced apoptosis in PC12 cells together with an alteration of the lipidic profile of the DRMs regarding their composition on phosphatidylinositol, phosphatidylcholine, and sphingomyelin [161]. Concerning changes of lipids in membrane rafts, a recent study revealed that electronic cigarette smoke in A549 provoked alterations in the levels of phosphatidylcholines, phosphatidylserine, and sphingomyelin, among others, in lipid raft fractions [21]. Moreover, the addition of nicotine exacerbated the differences in lipid raft composition [21]. 

### 4.3. Alterations in Rafts’ Proteome and Associated Cellular Effects

Following the rationale of the previous section for the effects of environmental toxicants on the lipid composition of lipid rafts, in this section, the evidence for the effect of environmental toxicants on the protein composition of lipid rafts will be presented together with associated cellular effects.

#### 4.3.1. Alterations in Lipid Raft-Associated Proteins

A recent study using red blood cells reported that mercury exposure leads to alterations in flotillin-2 levels, detected by electrophoresis of total membrane proteins [23]. This is highly relevant because in these cells, the protein flotillin-2 is reported as one of the major protein components of lipid rafts [162]. Moreover, at the structural level, the authors propose that mercury exposure could provoke membrane fragmentation of red blood cells [23], since flotillin-2 is responsible for membrane stability and is recognized to facilitate the association of protein complexes at the interface between membrane and cytosol [163]. Interestingly, exposure to copper was also described as having an effect on the distribution of flotillin-2 in the lipid rafts of neuroblastoma cells [89].

Another example of alterations in rafts’ proteomes is the displacement of proteins from these membrane regions, as occurs for occluding in human brain endothelial cells exposed to the industrial chemical 2,2’,4,4’,5,5’-hexachlorobiphenyl, also known as PCB153 [164]. The cellular mechanism by which this displacement occurs is related to PCB153-induced dephosphorylation of the threonine residues of occluding via activation of protein phosphatase 2A (PP2A) and MMP-2 [164]. In this cell model, MMP-2 activity was detected in lipid rafts and increased after PCB153 incubation. More importantly, MMP-2 inhibition prevented PCB153-induced loss of occluding from tight junctions and disruption of lipid raft assembly associated with endothelial barrier function (Figure 3). Other polychlorinated chemicals induce MMP-2 release by prostate cancer cells [156].

Exposure of CHO-K1 cells transfected with TSHR to the pesticide DDT abrogated the co-localization between the TSHR and lipid rafts, with consequences for receptor internalization [19]. Interestingly, the actions of DDT were not unspecific, as a lipophilic compound structurally related to DDT (diphenylethylene) did not mimic DDT’s effects. Also, the exposure of macrophages to B[a]P was reported to provoke a displacement of the CD32 protein from lipid rafts to non-lipid raft fractions, affecting macrophage effector functions [15] (Figure 3). Other authors showed that secondhand smoke depleted the expression of the cystic fibrosis transmembrane conductance regulator and lipid-raft-associated activity, a potential mechanism suggested for impaired phagocytosis [165]. Similar effects were observed for the SH-SY5Y human neuroblastoma cells incubated with copper ions, where a decrease of the γ-secretase protein in lipid rafts and activity was observed [89]. 

#### 4.3.2. Recruitment and Aggregation of Proteins in Lipid Rafts

Recently, exposure to vanadium (IV) compounds was revealed to promote aggregation and accumulation of the luteinizing hormone receptors in the plasma membrane of CHO cells, with the formation of clusters containing these receptors [119]. One of the compounds—bis(maltolato)oxovanadium(IV)—also provoked lipid packing. Indeed, exposure to these compounds was previously reported to produce similar effects in RBL-2H3 cells regarding lipid packing and insulin receptors [166]. 

The exposure to traffic-related PM revealed that it altered the processing of amyloid precursor protein in lipid raft fractions from in vivo and in vitro samples [17]. On hepatoma Hepa1c1c7 cells, the effect of B[a]P—a cigarette smoke and PM component—provoked changes in the membrane localization of flotillin-1 and the displacement of connexin-2 from lipid rafts [149]. Indeed, the recruitment of signaling proteins into lipid rafts after exposure to cigarette smoke extract or PM is not a surprise. In human lung fibroblasts, the recruitment of the death-inducing signaling complex into lipid rafts was reported after exposure to cigarette smoke extract [167]. In human alveolar epithelial cells, exposure to tire debris organic extracts—described to contain PM10 and PM2.5 [168]—resulted in an increase in lipid rafts and caveolae markers flotillin-1, caveolin-1, and CD55 in the prepared DRMs [169]. Also in these fractions, an increase in the levels of HMOX1 was observed after exposure to these extracts [169], a protein already signaled in Figure 2. Interestingly, a decade later, in the same cell line—A549—similar effects were observed for exposure to cigarette smoke extract or e-cigarette vapor condensate. This exposure provoked an increment in the expression of the lipid raft proteins caveolin-1, -2, and flotillin-1 [170]. It was observed that exposure of A549 cells to cigarette extract promoted the recruitment of inflammation-related proteins—NLRP10 and NLRP12—into the lipid rafts, as well as the interaction of NLRP12 with caveolin-1 [170]. In addition, e-cigarette smoke with or without nicotine promoted the formation of protein complexes associated with inflammation containing TRL4, NOD-1, and caveolin-1 in the lipid rafts [21]. A study investigating the effect of nicotine on macrophages highlighted the increase in nicotinic acetylcholine receptor expression and accumulation in lipid rafts and the consequent activation of the NLRP3 inflammasome [56]. Moreover, the disruption of lipid rafts by methyl-β-cyclodextrin reduced the accumulation of these receptors in lipid rafts and inflammasome activation [56]. Having in mind that inflammatory signaling is a common response to environmental pollutants, the effects of airborne PM and organic pollutants on the dynamics of inflammasome proteins in lipid rafts deserve further investigation.

#### 4.3.3. Activation of Other Signaling Pathways

Lipid rafts were reported as a structural scenario for the PCB153-induced phosphorylation of JAK and Src kinases and upregulation of cell adhesion molecules (CAMs) that affect leukocyte infiltration in brain endothelial cells [16]. In this study, cholesterol depletion blocked the lipid raft-dependent NADPH oxidase/JAK/EGFR signaling mechanism triggered by PCB153. Nevertheless, silencing caveolin-1 didn’t affect the upregulation of CAMs, indicating that caveolae are not a condition for the PCB153-induced phosphorylation mechanisms [16]. Remarkably, occludin co-immunoprecipitated with PP2A and caveolin-1, and the displacement of occludin from DRMs and increased permeability of endothelial cells were substantially reduced when PP2A activity was inhibited [164]. Therefore, the Src/PP2A described in other settings [171] might participate in the early toxic actions of PCB153 at membrane rafts (Figure 3).

Lipid rafts are also implicated in the signaling pathway associated with the TCR by favoring the co-localization of the TCR, of the linker for activation of T cells (LAT), and of the LCK [172]. This last one is a protein of the Src family that, after stimulation of TCR, is activated and favors a phosphorylation cascade involving ZAP70 and LAT, which, as a consequence, recruits PLC to rafts [173]. In this case, exposure of T lymphocytes to ethanol resulted in an inhibition of the co-localization of LCK, ZAP70, LAT, and PLC in plasma membrane lipid rafts essential for TCR signaling and consequent expression of interleukin-2 [173].

More recently, a study reinforced the role of G protein-coupled receptor (GPCR) activation by toxicants through lipid raft regions. In CHO cells, an increase in intracellular cAMP was observed after exposure to vanadium compounds, which is complementary to the aggregation of luteinizing hormone receptors in clusters, as described previously [119]. In addition to the luteinizing hormone receptor and TSHR already mentioned, there are GPCRs for a variety of peptide hormones, lipid mediators of inflammation, and biogenic amines (e.g., adrenaline), among other ligands, and GPCRs are commonly palmitoylated and located at cholesterol-rich domains of the plasma membrane [174,175]. In this regard, the β-adrenoceptor has been implicated in the PAH-induced increase of intracellular calcium concentration [176], and the beta-blocker carvedilol attenuates B[a]P toxicity [177]. GPCRs transduce the signal primarily by the cAMP/protein kinase A and the phosphatidylinositol/PLC/PKC pathways, incorporating calcium/calmodulin, ROS, and nitric oxide signaling, so their possible modulation by environmental toxicants warrants more extensive studies.

## 5. Conclusions and Future Perspectives

The evidence collected throughout this work points to the role of lipid rafts as targets of environmental toxicants—such as PM, PAHs, PCBs, pesticides, and metal ions, among others—unleashing cell signaling mechanisms consequent to toxic exposure. The identified triggering mechanisms may be toxicant-specific, while other mechanisms are common to different toxicants. Considering this, some suggestions for future research are given:

- When studying the effect of a toxicant in lipid rafts, several effects can be considered for research since they have already been reported to be common to different toxicant exposures. They include alteration of lipid raft composition (lipids and proteins), alteration of cholesterol content and membrane fluidity, recruitment or displacement of proteins from these membrane domains, and oxidative stress.
- The accumulation of toxicants in lipid rafts is not a clear point, with only PCB77 being reported to accumulate in these membrane domains [102]. Nevertheless, if similar behavior could be demonstrated for additional toxicants, it would strengthen the relevance of these lipid structures in environmental toxicology.
- The effect of environmental toxicants on inflammatory processes deserves to be further investigated, considering that inflammatory signaling is a common response to environmental pollutants [5], with some responses dependent on inflammarafts [101], and activation of inflammasomes [56].
- In the case of exposure to B[a]P, alteration of GM1 and raft protein localization is reported. With this in mind, investigating the effect of other environmental toxicants on the distribution of GM1 can be highly interesting if we consider that the location of GM1 near membrane channels affects their activity [67,68]. Moreover, the effect of B[a]P on the relocation of NHE-1 outside lipid rafts, with consequences for its apoptotic function [151], may be translated to other toxicants.
- The activation of a specific GPCR type via membrane clustering after exposure to vanadium [119], together with the wide range of ligands binding to these receptors and the vast signaling associated, makes these receptors a potential trigger for environmental toxicants, but whether this depends on lipid raft structure is a hypothesis that deserves to be studied.
- Another research dimension to be explored is how environmental toxicants can interfere with the interplay of different signals coming from proteins associated with lipid rafts. For example, the lipid rafts are near enzymatic systems producing ROS, like NADPH oxidases and nitric oxide synthases [16,66,78]. This gains even more relevance if we consider that different environmental toxicants are ligands of AhR, leading to activation of membrane NADPH oxidases [4,141].
- AhR is a reported target of PCBs, PAHs, PM, and persistent organic pollutants [140,141,146] and the non-canonical AhR signaling pathway [14,141,142] deserves to be explored for the effects of additional environmental toxicants. More specific data is needed to understand how membrane rafts/caveola modulate the activity of transcription factors like AhR and Nrf2, which are highly implicated in the cellular effects of environmental toxicants.
- Finally, to address the role of lipid rafts in triggering a specific cellular mechanism after any toxic exposure, it is important to employ different cell models expressing different membrane receptors and signaling components at lipid rafts since signaling differences were identified in this work. For example, in endothelial cells, the toxicant effects described to be associated with lipid rafts are changes in Nrf2 signaling, an increase in MCP-1 associated with AhR, p38, and JNK signaling pathways, and the displacement of occludin from lipid rafts is also described [140,145,164]. Nevertheless, in neurons, the involvement of lipid rafts in oxidative stress and the increase of cell calcium are potential cellular mechanisms affected by exposure to harmful compounds [13,17,77].

Information on the effect of environmental toxicants in lipid rafts and associated cell signaling mechanisms is compiled in this article, and to the best of our knowledge, this is the first review approaching the effect of environmental toxicants on lipid raft signaling. The collected data endorse that membrane raft-like domains can function as hubs for the regulation of toxic cellular mechanisms triggered by these harmful chemical agents. Therefore, this concept can provide meaningful insights into the comprehension of environmental toxicants’ molecular effects at the cellular level.

## Data Availability

Data is contained within the article.

## References

[1] Simons K., Toomre D. (2000). Lipid Rafts and Signal Transduction. Nat. Rev. Mol. Cell Biol..

[2] Placidi G., Campa C.C. (2021). Deliver on Time or Pay the Fine: Scheduling in Membrane Trafficking. Int. J. Mol. Sci..

[3] Greenlee J.D., Subramanian T., Liu K., King M.R. (2021). Rafting down the Metastatic Cascade: The Role of Lipid Rafts in Cancer Metastasis, Cell Death, and Clinical Outcomes. Cancer Res..

[4] Lagoa R., Marques-da-Silva D., Diniz M., Daglia M., Bishayee A. (2022). Molecular Mechanisms Linking Environmental Toxicants to Cancer Development: Significance for Protective Interventions with Polyphenols. Semin. Cancer Biol..

[5] Marques-da-Silva D., Videira P.A., Lagoa R. (2022). Registered Human Trials Addressing Environmental and Occupational Toxicant Exposures: Scoping Review of Immunological Markers and Protective Strategies. Environ. Toxicol. Pharmacol..

[6] Aminov Z., Haase R.F., Pavuk M., Carpenter D.O. (2013). Analysis of the Effects of Exposure to Polychlorinated Biphenyls and Chlorinated Pesticides on Serum Lipid Levels in Residents of Anniston, Alabama. Environ. Health.

[7] Adetona A.M., Adetona O., Gogal R.M., Diaz-Sanchez D., Rathbun S.L., Naeher L.P. (2017). Impact of Work Task-Related Acute Occupational Smoke Exposures on Select Proinflammatory Immune Parameters in Wildland Firefighters. J. Occup. Environ. Med..

[8] Goyal T., Mitra P., Singh P., Ghosh R., Sharma S., Sharma P. (2021). Association of MicroRNA Expression with Changes in Immune Markers in Workers with Cadmium Exposure. Chemosphere.

[9] Parks C.G., Santos A.d.S.E., Lerro C.C., DellaValle C.T., Ward M.H., Alavanja M.C., Berndt S.I., Beane Freeman L.E., Sandler D.P., Hofmann J.N. (2019). Lifetime Pesticide Use and Antinuclear Antibodies in Male Farmers from the Agricultural Health Study. Front. Immunol..

[10] Rider C.F., Carlsten C. (2019). Air Pollution and DNA Methylation: Effects of Exposure in Humans. Clin. Epigenetics.

[11] Rubini E., Minacori M., Paglia G., Macone A., Chichiarelli S., Altieri F., Eufemi M. (2021). Tomato and Olive Bioactive Compounds: A Natural Shield against the Cellular Effects Induced by β-Hexachlorocyclohexane-Activated Signaling Pathways. Molecules.

[12] Renu K., Mukherjee A.G., Wanjari U.R., Vinayagam S., Veeraraghavan V.P., Vellingiri B., George A., Lagoa R., Sattu K., Dey A. (2022). Misuse of Cardiac Lipid upon Exposure to Toxic Trace Elements—A Focused Review. Molecules.

[13] Fortalezas S., Marques-da-Silva D., Gutierrez-Merino C. (2018). Creatine Protects Against Cytosolic Calcium Dysregulation, Mitochondrial Depolarization and Increase of Reactive Oxygen Species Production in Rotenone-Induced Cell Death of Cerebellar Granule Neurons. Neurotox. Res..

[14] Brinchmann B.C., Le Ferrec E., Podechard N., Lagadic-Gossmann D., Shoji K.F., Penna A., Kukowski K. (2018). Lipophilic Chemicals from Diesel Exhaust Particles Trigger Calcium Response in Human Endothelial Cells via Aryl Hydrocarbon Receptor Non-Genomic Signalling. Int. J. Mol. Sci..

[15] Clark R.S., Pellom S.T., Booker B., Ramesh A., Zhang T., Shanker A., Maguire M., Juarez P.D., Patricia M.J., Langston M.A. (2016). Validation of Research Trajectory 1 of an Exposome Framework: Exposure to Benzo(a)Pyrene Confers Enhanced Susceptibility to Bacterial Infection. Environ. Res..

[16] Eum S.Y., Andras I., Hennig B., Toborek M. (2009). NADPH Oxidase and Lipid Raft-Associated Redox Signaling Are Required for PCB153-Induced Upregulation of Cell Adhesion Molecules in Human Brain Endothelial Cells. Toxicol. Appl. Pharmacol..

[17] Cacciottolo M., Morgan T.E., Saffari A.A., Shirmohammadi F., Forman H.J., Sioutas C., Finch C.E. (2020). Traffic-Related Air Pollutants (TRAP-PM) Promote Neuronal Amyloidogenesis through Oxidative Damage to Lipid Rafts. Free Radic. Biol. Med..

[18] Tekpli X., Rissel M., Huc L., Catheline D., Sergent O., Rioux V., Legrand P., Holme J.A., Dimanche-Boitrel M.T., Lagadic-Gossmann D. (2010). Membrane Remodeling, an Early Event in Benzo[α]Pyrene-Induced Apoptosis. Toxicol. Appl. Pharmacol..

[19] De Gregorio F., Pellegrino M., Picchietti S., Belardinelli M.C., Taddei A.R., Fausto A.M., Rossi M., Maggio R., Giorgi F. (2011). The Insecticide 1,1,1-Trichloro-2,2-Bis(p-Chlorophenyl) Ethane (DDT) Alters the Membrane Raft Location of the TSH Receptor Stably Expressed in Chinese Hamster Ovary Cells. Toxicol. Appl. Pharmacol..

[20] Xu Y., Wang W., Zhou J., Chen M., Huang X., Zhu Y., Xie X., Li W., Zhang Y., Kan H. (2019). Metabolomics Analysis of a Mouse Model for Chronic Exposure to Ambient PM2.5. Environ. Pollut..

[21] Singh D.P., Begum R., Kaur G., Bagam P., Kambiranda D., Singh R., Batra S. (2021). E-Cig Vapor Condensate Alters Proteome and Lipid Profiles of Membrane Rafts: Impact on Inflammatory Responses in A549 Cells. Cell Biol. Toxicol..

[22] Bhardwaj P., Kumar M., Dhatwalia S.K., Garg M.L., Dhawan D.K. (2019). Acetyl-11-Keto-β-Boswellic Acid Modulates Membrane Dynamics in Benzo(a)Pyrene-Induced Lung Carcinogenesis. Mol. Cell. Biochem..

[23] Notariale R., Längst E., Perrone P., Crettaz D., Prudent M., Manna C. (2022). Effect of Mercury on Membrane Proteins Anionic Transport and Cell Morphology in Human Erythrocytes. Cell. Physiol. Biochem..

[24] Brown D.A., London E. (1998). Structure and Origin of Ordered Lipid Domains in Biological Membranes. J. Membr. Biol..

[25] Hjort Ipsen J., Karlström G., Mourtisen O.G., Wennerström H., Zuckermann M.J. (1987). Phase Equilibria in the Phosphatidylcholine-Cholesterol System. BBA—Biomembr..

[26] Pike L.J. (2003). Lipid Rafts: Bringing Order to Chaos. J. Lipid Res..

[27] Simons K., Ikonen E. (1997). Functional Rafts in Cell Membranes. Nature.

[28] Waheed A.A., Freed E.O., Parent L.J. (2018). The Role of Lipids in Retroviral Replication. Retrovirus-Cell Interactions.

[29] Shankar J., Boscher C., Nabi I.R. (2015). Caveolin-1, Galectin-3 and Lipid Raft Domains in Cancer Cell Signalling. Essays Biochem..

[30] Bartlett K., Kim K. (2014). Insight into Tor2, a Budding Yeast Microdomain Protein. Eur. J. Cell Biol..

[31] Krapf D. (2018). Compartmentalization of the Plasma Membrane. Curr. Opin. Cell Biol..

[32] Godoy V., Riquelme G. (2008). Distinct Lipid Rafts in Subdomains from Human Placental Apical Syncytiotrophoblast Membranes. J. Membr. Biol..

[33] Toledo A., Huang Z., Coleman J.L., London E., Benach J.L. (2018). Lipid Rafts Can Form in the Inner and Outer Membranes of Borrelia Burgdorferi and Have Different Properties and Associated Proteins. Mol. Microbiol..

[34] Blouin C.M., Prado C., Takane K.K., Lasnier F., Garcia-Ocana A., Ferré P., Dugail I., Hajduch E. (2010). Plasma Membrane Subdomain Compartmentalization Contributes to Distinct Mechanisms of Ceramide Action on Insulin Signaling. Diabetes.

[35] Parton R.G. (2018). Caveolae: Structure, Function, and Relationship to Disease. Annu. Rev. Cell Dev. Biol..

[36] Yamada E. (1955). The Fine Structure of the Fall Bladder Epithelium of the Mouse. J. Biophys. Biochem. Cytol..

[37] Razani B., Woodman S.E., Lisanti M.P. (2002). Caveolae: From Cell Biology to Animal Physiology. Pharmacol. Rev..

[38] Martinez-Outschoorn U.E., Sotgia F., Lisanti M.P. (2015). Caveolae and Signalling in Cancer. Nat. Rev. Cancer.

[39] Hansen C.G., Nichols B.J. (2010). Exploring the Caves: Cavins, Caveolins and Caveolae. Trends Cell Biol..

[40] Filippini A., D’alessio A. (2020). Caveolae and Lipid Rafts in Endothelium: Valuable Organelles for Multiple Functions. Biomolecules.

[41] Parton R.G., Tillu V.A., Collins B.M. (2018). Caveolae. Curr. Biol..

[42] Sevcsik E., Schütz G.J. (2016). With or without Rafts? Alternative Views on Cell Membranes. BioEssays.

[43] Levental I., Levental K.R., Heberle F.A. (2020). Lipid Rafts: Controversies Resolved, Mysteries Remain. Trends Cell Biol..

[44] Klotzsch E., Schütz G.J. (2013). A Critical Survey of Methods to Detect Plasma Membrane Rafts. Philos. Trans. R. Soc. B Biol. Sci..

[45] Suzuki K.G.N., Kusumi A. (2023). Refinement of Singer-Nicolson Fluid-Mosaic Model by Microscopy Imaging: Lipid Rafts and Actin-Induced Membrane Compartmentalization. Biochim. Biophys. Acta—Biomembr..

[46] Kusumi A., Fujiwara T.K., Tsunoyama T.A., Kasai R.S., Liu A.A., Hirosawa K.M., Kinoshita M., Matsumori N., Komura N., Ando H. (2020). Defining Raft Domains in the Plasma Membrane. Traffic.

[47] Regen S.L. (2020). The Origin of Lipid Rafts. Biochemistry.

[48] Simons K., Ehehalt R. (2002). Cholesterol, Lipid Rafts, and Disease. J. Clin. Investig..

[49] Kraft M.L. (2017). Sphingolipid Organization in the Plasma Membrane and the Mechanisms That Influence It. Front. Cell Dev. Biol..

[50] Simons K., Sampaio J.L. (2011). Membrane Organization and Lipid Rafts. Cold Spring Harb. Perspect. Biol..

[51] Chang W.J., Rothberg K.G., Kamen B.A., Anderson R.G.W. (1992). Lowering the Cholesterol Content of MA104 Cells Inhibits Receptor-Mediated Transport of Folate. J. Cell Biol..

[52] Ouweneel A.B., Thomas M.J., Sorci-Thomas M.G. (2020). The Ins and Outs of Lipid Rafts: Functions in Intracellular Cholesterol Homeostasis, Microparticles, and Cell Membranes. J. Lipid Res..

[53] Fessler M.B., Parks J.S. (2011). Intracellular Lipid Flux and Membrane Microdomains as Organizing Principles in Inflammatory Cell Signaling. J. Immunol..

[54] Fadeyibi O., Rybalchenko N., Mabry S., Nguyen D.H., Cunningham R.L. (2022). The Role of Lipid Rafts and Membrane Androgen Receptors in Androgen’s Neurotoxic Effects. J. Endocr. Soc..

[55] Premasekharan G., Nguyen K., Contreras J., Ramon V., Leppert V.J., Forman H.J. (2011). Iron-Mediated Lipid Peroxidation and Lipid Raft Disruption in Low-Dose Silica-Induced Macrophage Cytokine Production. Free Radic. Biol. Med..

[56] Duan F., Zeng C., Liu S., Gong J., Hu J., Li H., Tan H. (2021). A1-NAchR-Mediated Signaling Through Lipid Raft Is Required for Nicotine-Induced NLRP3 Inflammasome Activation and Nicotine-Accelerated Atherosclerosis. Front. Cell Dev. Biol..

[57] Liu S., Tao J., Duan F., Li H., Tan H. (2022). HHcy Induces Pyroptosis and Atherosclerosis via the Lipid Raft-Mediated NOX-ROS-NLRP3 Inflammasome Pathway in ApoE^−/−^ Mice. Cells.

[58] Fortalezas S., Marques-da-Silva D., Gutierrez-Merino C. (2018). Methyl-β-Cyclodextrin Impairs the Phosphorylation of the Β2 Subunit of L-Type Calcium Channels and Cytosolic Calcium Homeostasis in Mature Cerebellar Granule Neurons. Int. J. Mol. Sci..

[59] Dietrich C., Bagatolli L.A., Volovyk Z.N., Thompson N.L., Levi M., Jacobson K., Gratton E. (2001). Lipid Rafts Reconstituted in Model Membranes. Biophys. J..

[60] Palestini P., Calvi C., Conforti E., Daffara R., Botto L., Miserocchi G. (2003). Compositional Changes in Lipid Microdomains of Air-Blood Barrier Plasma Membranes in Pulmonary Interstitial Edema. J. Appl. Physiol..

[61] Ledeen R.W., Wu G. (2015). The Multi-Tasked Life of GM1 Ganglioside, a True Factotum of Nature. Trends Biochem. Sci..

[62] Van Heyningen S. (1974). Cholera Toxin: Interaction of Subunits with Ganglioside GM1. Science.

[63] Day C.A., Kenworthy A.K. (2015). Functions of Cholera Toxin B-Subunit as a Raft Cross-Linker. Essays Biochem..

[64] Kenworthy A.K., Schmieder S.S., Raghunathan K., Tiwari A., Wang T., Kelly C.V., Lencer W.I. (2021). Cholera Toxin as a Probe for Membrane Biology. Toxins.

[65] Samhan-Arias A.K., López-Sánchez C., Marques-da-Silva D., Lagoa R., Garcia-Lopez V., García-Martínez V., Gutierrez-Merino C. (2016). High Expression of Cytochrome b_5_ Reductase Isoform 3/Cytochrome b_5_ System in the Cerebellum and Pyramidal Neurons of Adult Rat Brain. Brain Struct. Funct..

[66] Samhan-Arias A.K., Garcia-Bereguiain M.A., Martin-Romero F.J., Gutierrez-Merino C. (2009). Clustering of Plasma Membrane-Bound Cytochrome B5 Reductase within “lipid Raft” Microdomains of the Neuronal Plasma Membrane. Mol. Cell. Neurosci..

[67] Puljko B., Stojanović M., Ilic K., Kalanj-Bognar S., Mlinac-Jerkovic K. (2022). Start Me Up: How Can Surrounding Gangliosides Affect Sodium-Potassium ATPase Activity and Steer towards Pathological Ion Imbalance in Neurons?. Biomedicines.

[68] Ilic K., Lin X., Malci A., Stojanović M., Puljko B., Rožman M., Vukelić Ž., Heffer M., Montag D., Schnaar R.L. (2021). Plasma Membrane Calcium ATPase-Neuroplastin Complexes Are Selectively Stabilized in GM1-Containing Lipid Rafts. Int. J. Mol. Sci..

[69] Lucero H.A., Robbins P.W. (2004). Lipid Rafts-Protein Association and the Regulation of Protein Activity. Arch. Biochem. Biophys..

[70] Kurzchalia T.V., Parton R.G. (1999). Membrane Microdomains and Caveolae. Curr. Opin. Cell Biol..

[71] Galbiati F., Razani B., Lisanti M.P. (2001). Emerging Themes in Lipid Rafts and Caveolae. Cell.

[72] Vassilieva E.V., Ivanov A.I., Nusrat A. (2009). Flotillin-1 Stabilizes Caveolin-1 in Intestinal Epithelial Cells. Biochem. Biophys. Res. Commun..

[73] Foster L.J., De Hoog C.L., Mann M. (2003). Unbiased Quantitative Proteomics of Lipid Rafts Reveals High Specificity for Signaling Factors. Proc. Natl. Acad. Sci. USA.

[74] Magee A.I., Parmryd I. (2003). Detergent-Resistant Membranes and the Protein Composition of Lipid Rafts. Genome Biol..

[75] Marques-da-Silva D., Samhan-Arias A.K., Tiago T., Gutierrez-Merino C. (2010). L-Type Calcium Channels and Cytochrome B_5_ Reductase Are Components of Protein Complexes Tightly Associated with Lipid Rafts Microdomains of the Neuronal Plasma Membrane. J. Proteom..

[76] Marques-da-Silva D., Gutierrez-Merino C. (2012). L-Type Voltage-Operated Calcium Channels, N-Methyl-d-Aspartate Receptors and Neuronal Nitric-Oxide Synthase Form a Calcium/Redox Nano-Transducer within Lipid Rafts. Biochem. Biophys. Res. Commun..

[77] Marques-da-Silva D., Gutierrez-Merino C. (2014). Caveolin-Rich Lipid Rafts of the Plasma Membrane of Mature Cerebellar Granule Neurons Are Microcompartments for Calcium/Reactive Oxygen and Nitrogen Species Cross-Talk Signaling. Cell Calcium.

[78] Samhan-Arias A.K., Marques-da-Silva D., Yanamala N., Gutierrez-Merino C. (2012). Stimulation and Clustering of Cytochrome b 5 Reductase in Caveolin-Rich Lipid Microdomains Is an Early Event in Oxidative Stress-Mediated Apoptosis of Cerebellar Granule Neurons. J. Proteom..

[79] Fortalezas S., Poejo J., Samhan-Arias A.K., Gutierrez-Merino C. (2019). Cholesterol-Rich Plasma Membrane Submicrodomains Can Be a Major Extramitochondrial Source of Reactive Oxygen Species in Partially Depolarized Mature Cerebellar Granule Neurons in Culture. J. Neurophysiol. Neurol. Disord..

[80] Tiago T., Palma P.S., Gutierrez-Merino C., Aureliano M. (2010). Peroxynitrite-Mediated Oxidative Modifications of Myosin and Implications on Structure and Function. Free Radic. Res..

[81] Gupta N., DeFranco A.L. (2007). Lipid Rafts and B Cell Signaling. Semin. Cell Dev. Biol..

[82] Delos Santos R.C., Garay C., Antonescu C.N. (2015). Charming Neighborhoods on the Cell Surface: Plasma Membrane Microdomains Regulate Receptor Tyrosine Kinase Signaling. Cell. Signal..

[83] Janes P.W., Ley S.C., Magee A.I., Kabouridis P.S. (2000). The Role of Lipid Rafts in T Cell Antigen Receptor (TCR) Signalling. Semin. Immunol..

[84] Maselli A., Pierdominici M., Vitale C., Ortona E. (2015). Membrane Lipid Rafts and Estrogenic Signalling: A Functional Role in the Modulation of Cell Homeostasis. Apoptosis.

[85] Li B., Qin Y., Yu X., Xu X., Yu W. (2022). Lipid Raft Involvement in Signal Transduction in Cancer Cell Survival, Cell Death and Metastasis. Cell Prolif..

[86] Mollinedo F., Gajate C. (2020). Lipid Rafts as Signaling Hubs in Cancer Cell Survival/Death and Invasion: Implications in Tumor Progression and Therapy. J. Lipid Res..

[87] Marin R., Nils T., Sten J. (2018). Lipid Rafts as Molecular Platforms of Neuronal Toxicity and Survival: Two Sides of the Same Coin. Lipid Rafts: Properties and Role in Signaling.

[88] Moll T., Marshall J.N.G., Soni N., Zhang S., Cooper-Knock J., Shaw P.J. (2021). Membrane Lipid Raft Homeostasis Is Directly Linked to Neurodegeneration. Essays Biochem..

[89] Hung Y.H., Robb E.L., Voltakis I., Ho M., Evin G., Li Q.X., Culvenor J.G., Masters C.L., Cherny R.A., Bush A.I. (2009). Paradoxical Condensation of Copper with Elevated β-Amyloid in Lipid Rafts under Cellular Copper Deficiency Conditions. Implications for Alzheimer Disease. J. Biol. Chem..

[90] Suzuki T., Zhang J., Miyazawa S., Liu Q., Farzan M.R., Yao W.D. (2011). Association of Membrane Rafts and Postsynaptic Density: Proteomics, Biochemical, and Ultrastructural Analyses. J. Neurochem..

[91] Eid A., Mhatre-Winters I., Sammoura F.M., Edler M.K., von Stein R., Hossain M.M., Han Y., Lisci M., Carney K., Konsolaki M. (2022). Effects of DDT on Amyloid Precursor Protein Levels and Amyloid Beta Pathology: Mechanistic Links to Alzheimer’s Disease Risk. Environ. Health Perspect..

[92] Morris G., Walder K., Puri B.K., Berk M., Maes M. (2016). The Deleterious Effects of Oxidative and Nitrosative Stress on Palmitoylation, Membrane Lipid Rafts and Lipid-Based Cellular Signalling: New Drug Targets in Neuroimmune Disorders. Mol. Neurobiol..

[93] Evangelisti E., Wright D., Zampagni M., Cascella R., Fiorillo C., Bagnoli S., Relini A., Nichino D., Scartabelli T., Nacmias B. (2013). Lipid Rafts Mediate Amyloid-Induced Calcium Dyshomeostasis and Oxidative Stress in Alzheimer’s Disease. Curr. Alzheimer Res..

[94] Poejo J., Salazar J., Mata A.M., Gutierrez-merino C. (2021). Binding of Amyloid β(1–42)-calmodulin Complexes to Plasma Membrane Lipid Rafts in Cerebellar Granule Neurons Alters Resting Cytosolic Calcium Homeostasis. Int. J. Mol. Sci..

[95] Mattson M.P., Chan S.L. (2001). Dysregulation of Cellular Calcium Homeostasis in Alzheimer’s Disease: Bad Genes and Bad Habits. J. Mol. Neurosci..

[96] Sattler R., Tymianski M. (2000). Molecular Mechanisms of Calcium-Dependent Excitotoxicity. J. Mol. Med..

[97] Choi D.W. (1995). Calcium: Still Center-Stage in Hypoxic-Ischemic Neuronal Death. Trends Neurosci..

[98] Jiang L., Fernandes D., Mehta N., Bean J.L., Michaelis M.L., Zaidi A. (2007). Partitioning of the Plasma Membrane Ca^2+^-ATPase into Lipid Rafts in Primary Neurons: Effects of Cholesterol Depletion. J. Neurochem..

[99] Duan W., Zhou J., Li W., Zhou T., Chen Q., Yang F., Wei T. (2013). Plasma Membrane Calcium ATPase 4b Inhibits Nitric Oxide Generation through Calcium-Induced Dynamic Interaction with Neuronal Nitric Oxide Synthase. Protein Cell.

[100] Legler D.F., Micheau O., Doucey M.A., Tschopp J., Bron C. (2003). Recruitment of TNF Receptor 1 to Lipid Rafts Is Essential for TNFα-Mediated NF-ΚB Activation. Immunity.

[101] Miller Y.I., Navia-Pelaez J.M., Corr M., Yaksh T.L. (2020). Lipid Rafts in Glial Cells: Role in Neuroinflammation and Pain Processing. J. Lipid Res..

[102] Lim E.J., Májková Z., Xu S., Bachas L., Arzuaga X., Smart E., Tseng M.T., Toborek M., Hennig B. (2008). Coplanar Polychlorinated Biphenyl-Induced CYP1A1 Is Regulated through Caveolae Signaling in Vascular Endothelial Cells. Chem. Biol. Interact..

[103] Shihata W.A., Michell D.L., Andrews K.L., Chin-Dusting J.P.F. (2016). Caveolae: A Role in Endothelial Inflammation and Mechanotransduction?. Front. Physiol..

[104] Sorci-Thomas M.G., Thomas M.J. (2016). Microdomains, Inflammation, and Atherosclerosis. Circ. Res..

[105] Navia-Pelaez J.M., Agatisa-Boyle C., Choi S.-H., Sak Kim Y., Li S., Alekseeva E., Weldy K., Miller Y.I. (2023). Differential Expression of Inflammarafts in Macrophage Foam Cells and in Nonfoamy Macrophages in Atherosclerotic Lesions. Arterioscler. Thromb. Vasc. Biol..

[106] Vieth J.A., Kim M.K., Glaser D., Stiles K., Schreiber A.D., Worth R.G. (2013). FcγRIIa Requires Lipid Rafts, but Not Co-Localization into Rafts, for Effector Function. Inflamm. Res..

[107] Scheel-Toellner D., Wang K., Singh R., Majeed S., Raza K., Curnow S.J., Salmon M., Lord J.M. (2002). The Death-Inducing Signalling Complex Is Recruited to Lipid Rafts in Fas-Induced Apoptosis. Biochem. Biophys. Res. Commun..

[108] Gajate C., Gonzalez-Camacho F., Mollinedo F. (2009). Involvement of Raft Aggregates Enriched in Fas/CD95 Death-Inducing Signaling Complex in the Antileukemic Action of Edelfosine in Jurkat Cells. PLoS ONE.

[109] Molnár E., Swamy M., Holzer M., Beck-García K., Worch R., Thiele C., Guigas G., Boye K., Luescher I.F., Schwille P. (2012). Cholesterol and Sphingomyelin Drive Ligand-Independent T-Cell Antigen Receptor Nanoclustering. J. Biol. Chem..

[110] Miguel L., Owen D.M., Lim C., Liebig C., Evans J., Magee A.I., Jury E.C. (2011). Primary Human CD4 + T Cells Have Diverse Levels of Membrane Lipid Order That Correlate with Their Function. J. Immunol..

[111] Jury E.C., Kabouridis P.S., Flores-Borja F., Mageed R.A., Isenberg D.A. (2004). Altered Lipid Raft–Associated Signaling and Ganglioside Expression in T Lymphocytes from Patients with Systemic Lupus Erythematosus. J. Clin. Investig..

[112] Krishnan S., Nambiar M.P., Warke V.G., Fisher C.U., Mitchell J., Delaney N., Tsokos G.C. (2004). Alterations in Lipid Raft Composition and Dynamics Contribute to Abnormal T Cell Responses in Systemic Lupus Erythematosus. J. Immunol..

[113] Flores-Borja F., Kabouridis P.S., Jury E.C., Isenberg D.A., Mageed R.A. (2007). Altered Lipid Raft-Associated Proximal Signaling and Translocation of CD45 Tyrosine Phosphatase in B Lymphocytes from Patients with Systemic Lupus Erythematosus. Arthritis Rheum..

[114] Sengupta S., Karsalia R., Morrissey A., Bamezai A.K. (2021). Cholesterol-Dependent Plasma Membrane Order (Lo) Is Critical for Antigen-Specific Clonal Expansion of CD4+ T Cells. Sci. Rep..

[115] Saeki K., Miura Y., Aki D., Kurosaki T., Yoshimura A. (2003). The B Cell-Specific Major Raft Protein, Raftlin, Is Necessary for the Integrity of Lipid Raft and BCR Signal Transduction. EMBO J..

[116] Kumbul Y.Ç., Yasan H., Okur E., Tüz M., Sivrice M.E., Akın V., Şirin F.B., Doğan Kıran E. (2022). The Role of Raftlin in the Pathogenesis of Chronic Rhinosinusitis with Nasal Polyps. Eur. Arch. Oto-Rhino-Laryngol..

[117] Latif R., Ando T., Davies T.F. (2007). Lipid Rafts Are Triage Centers for Multimeric and Monomeric Thyrotropin Receptor Regulation. Endocrinology.

[118] Smith S.M.L., Lei Y., Liu J., Cahill M.E., Hagen G.M., Barisas B.G., Roess D.A. (2006). Luteinizing Hormone Receptors Translocate to Plasma Membrane Microdomains after Binding of Human Chorionic Gonadotropin. Endocrinology.

[119] Althumairy D., Murakami H.A., Zhang D., Barisas B.G., Roess D.A., Crans D.C. (2020). Effects of Vanadium(IV) Compounds on Plasma Membrane Lipids Lead to G Protein-Coupled Receptor Signal Transduction. J. Inorg. Biochem..

[120] Marin R., Diaz M. (2018). Estrogen Interactions with Lipid Rafts Related to Neuroprotection. Impact of Brain Ageing and Menopause. Front. Neurosci..

[121] Canerina-Amaro A., Hernandez-Abad L.G., Ferrer I., Quinto-Alemany D., Mesa-Herrera F., Ferri C., Puertas-Avendaño R.A., Diaz M., Marin R. (2017). Lipid Raft ER Signalosome Malfunctions in Menopause and Alzheimer’s Disease. Front. Biosci.—Sch..

[122] Mesa-Herrera F., Marín R., Torrealba E., Santos G., Díaz M. (2022). Neuronal ER-Signalosome Proteins as Early Biomarkers in Prodromal Alzheimer’s Disease Independent of Amyloid-β Production and Tau Phosphorylation. Front. Mol. Neurosci..

[123] Marques-Da-Silva D., Rodrigues J.R., Lagoa R., de Oliveira M.R. (2021). Anthocyanins, Effects in Mitochondria and Metabolism. Mitochondrial Physiology and Vegetal Molecules Therapeutic Potential of Natural Compounds on Mitochondrial Health.

[124] Ghanbari-Movahed M., Shafiee S., Burcher J.T., Lagoa R., Farzaei M.H., Bishayee A. (2023). Anticancer Potential of Apigenin and Isovitexin with Focus on Oncogenic Metabolism in Cancer Stem Cells. Metabolites.

[125] Svensson K.J., Christianson H.C., Wittrup A., Bourseau-Guilmain E., Lindqvist E., Svensson L.M., Mörgelin M., Belting M. (2013). Exosome Uptake Depends on ERK1/2-Heat Shock Protein 27 Signaling and Lipid Raft-Mediated Endocytosis Negatively Regulated by Caveolin-1. J. Biol. Chem..

[126] Skryabin G.O., Komelkov A.V., Savelyeva E.E., Tchevkina E.M. (2020). Lipid Rafts in Exosome Biogenesis. Biochemistry.

[127] Schubert A.L., Schubert W., Spray D.C., Lisanti M.P. (2002). Connexin Family Members Target to Lipid Raft Domains and Interact with Caveolin-1. Biochemistry.

[128] Martins-Marques T., Ribeiro-Rodrigues T., Batista-Almeida D., Aasen T., Kwak B.R., Girao H. (2019). Biological Functions of Connexin43 Beyond Intercellular Communication. Trends Cell Biol..

[129] Tekpli X., Huc L., Lacroix J., Rissel M., Poët M., Noël J., Dimanche-Boitrel M.T., Counillon L., Lagadic-Gossmann D. (2008). Regulation of Na+/H+ Exchanger 1 Allosteric Balance by Its Localization in Cholesterol- and Caveolin-Rich Membrane Microdomains. J. Cell. Physiol..

[130] Casaburi I., Chimento A., De Luca A., Nocito M., Sculco S., Avena P., Trotta F., Rago V., Sirianni R., Pezzi V. (2018). Cholesterol as an Endogenous ERRα Agonist: A New Perspective to Cancer Treatment. Front. Endocrinol..

[131] Yamamoto Y., Tomiyama A., Sasaki N., Yamaguchi H., Shirakihara T., Nakashima K., Kumagai K., Takeuchi S., Toyooka T., Otani N. (2018). Intracellular Cholesterol Level Regulates Sensitivity of Glioblastoma Cells against Temozolomide-Induced Cell Death by Modulation of Caspase-8 Activation via Death Receptor 5-Accumulation and Activation in the Plasma Membrane Lipid Raft. Biochem. Biophys. Res. Commun..

[132] George K.S., Wu S. (2012). Lipid Raft: A Floating Island of Death or Survival. Toxicol. Appl. Pharmacol..

[133] Sikkema J., de Bont J.A., Poolman B. (1995). Mechanisms of Membrane Toxicity of Hydrocarbons. Microbiol. Rev..

[134] Buff K., Berndt J. (1981). Interaction of DDT (1,1,1-Trichloro-2,2-BIS(p-Chlorophenyl)-Ethane with Liposomal Phospholipids. BBA—Biomembr..

[135] Antunes-Madeira M.C., Madeira V.M.C. (1990). Membrane Fluidity as Affected by the Organochlorine Insecticide DDT. BBA—Biomembr..

[136] Endo S., Escher B.I., Goss K.U. (2011). Capacities of Membrane Lipids to Accumulate Neutral Organic Chemicals. Environ. Sci. Technol..

[137] Broniatowski M., Binczycka M., Wójcik A., Flasiński M., Wydro P. (2017). Polycyclic Aromatic Hydrocarbons in Model Bacterial Membranes—Langmuir Monolayer Studies. Biochim. Biophys. Acta—Biomembr..

[138] Yang H., Li H., Liu L., Zhou Y., Long X. (2019). Molecular Simulation Studies on the Interactions of 2,4,6-Trinitrotoluene and Its Metabolites with Lipid Membranes. J. Phys. Chem. B.

[139] Subuddhi U., Mishra A.K. (2006). Prototropism of 1-Hydroxypyrene in Liposome Suspensions: Implications towards Fluorescence Probing of Lipid Bilayers in Alkaline Medium. Photochem. Photobiol. Sci..

[140] Majkova Z., Smart E., Toborek M., Hennig B. (2009). Up-Regulation of Endothelial Monocyte Chemoattractant Protein-1 by Coplanar PCB77 Is Caveolin-1-Dependent. Toxicol. Appl. Pharmacol..

[141] Vogel C.F.A., Van Winkle L.S., Esser C., Haarmann-Stemmann T. (2020). The Aryl Hydrocarbon Receptor as a Target of Environmental Stressors—Implications for Pollution Mediated Stress and Inflammatory Responses. Redox Biol..

[142] Rey-Barroso J., Alvarez-Barrientos A., Rico-Leo E., Contador-Troca M., Carvajal-Gonzalez J.M., Echarri A., Del Pozo M.A., Fernandez-Salguero P.M. (2014). The Dioxin Receptor Modulates Caveolin-1 Mobilization during Directional Migration: Role of Cholesterol. Cell Commun. Signal..

[143] Hennig B., Reiterer G., Majkova Z., Oesterling E., Meerarani P., Toborek M. (2005). Modification of Environmental Toxicity by Nutrients. Implications in Atherosclerosis. Cardiovasc. Toxicol..

[144] Ramadass P., Meerarani P., Toborek M., Robertson L.W., Hennig B. (2003). Dietary Flavonoids Modulate PCB-Induced Oxidative Stress, CYP1A1 Induction, and AhR-DNA Binding Activity in Vascular Endothelial Cells. Toxicol. Sci..

[145] Petriello M.C., Han S.G., Newsome B.J., Hennig B. (2014). PCB 126 Toxicity Is Modulated by Cross-Talk between Caveolae and Nrf2 Signaling. Toxicol. Appl. Pharmacol..

[146] Oesterling E., Toborek M., Hennig B. (2008). Benzo[a]Pyrene Induces Intercellular Adhesion Molecule-1 through a Caveolae and Aryl Hydrocarbon Receptor Mediated Pathway. Toxicol. Appl. Pharmacol..

[147] Collin A., Hardonnière K., Chevanne M., Vuillemin J., Podechard N., Burel A., Dimanche-Boitrel M.T., Lagadic-Gossmann D., Sergent O. (2014). Cooperative Interaction of Benzo[a]Pyrene and Ethanol on Plasma Membrane Remodeling Is Responsible for Enhanced Oxidative Stress and Cell Death in Primary Rat Hepatocytes. Free Radic. Biol. Med..

[148] Gorria M., Tekpli X., Sergent O., Huc L., Gaboriau F., Rissel M., Chevanne M., Dimanche-Boitrel M.-T., Lagadic-Gossmann D. (2006). Membrane Fluidity Changes Are Associated with Benzo[a]Pyrene-Induced Apoptosis in F258 Cells: Protection by Exogenous Cholesterol. Ann. N. Y. Acad. Sci..

[149] Tekpli X., Rivedal E., Gorria M., Landvik N.E., Rissel M., Dimanche-Boitrel M.T., Baffet G., Holme J.A., Lagadic-Gossmann D. (2010). The B[a]P-Increased Intercellular Communication via Translocation of Connexin-43 into Gap Junctions Reduces Apoptosis. Toxicol. Appl. Pharmacol..

[150] Goldstein J.L., Brown M.S. (1990). Regulation of the Mevalonate Pathway. Nature.

[151] Tekpli X., Huc L., Sergent O., Dendelé B., Dimanche-Boitrel M.T., Holme J.A., Lagadic-Gossmann D. (2012). NHE-1 Relocation Outside Cholesterol-Rich Membrane Microdomains Is Associated with Its Benzo[a]Pyrene-Related Apoptotic Function. Cell. Physiol. Biochem..

[152] Dendelé B., Tekpli X., Hardonnière K., Holme J.A., Debure L., Catheline D., Arlt V.M., Nagy E., Phillips D.H., Øvrebø S. (2014). Protective Action of N-3 Fatty Acids on Benzo[a]Pyrene-Induced Apoptosis through the Plasma Membrane Remodeling-Dependent NHE1 Pathway. Chem. Biol. Interact..

[153] Bazzoni G.B., Bollini A.N., Hernández G.N., Contini M.D.C., Chiarotto M.M., Rasia M.L. (2005). In Vivo Effect of Aluminium upon the Physical Properties of the Erythrocyte Membrane. J. Inorg. Biochem..

[154] Yilmaz B., Sandal S., Chen C.H., Carpenter D.O. (2006). Effects of PCB 52 and PCB 77 on Cell Viability, [Ca^2+^] i Levels and Membrane Fluidity in Mouse Thymocytes. Toxicology.

[155] Tan Y. (2004). Ortho-Substituted but Not Coplanar PCBs Rapidly Kill Cerebellar Granule Cells. Toxicol. Sci..

[156] Bedia C., Dalmau N., Jaumot J., Tauler R. (2015). Phenotypic Malignant Changes and Untargeted Lipidomic Analysis of Long-Term Exposed Prostate Cancer Cells to Endocrine Disruptors. Environ. Res..

[157] Yun U.J., Lee J.H., Shim J., Yoon K., Goh S.H., Yi E.H., Ye S.K., Lee J.S., Lee H., Park J. (2019). Anti-Cancer Effect of Doxorubicin Is Mediated by Downregulation of HMG-Co A Reductase via Inhibition of EGFR/Src Pathway. Lab. Investig..

[158] Lagoa R., Gañán C., López-Sánchez C., García-Martínez V., Gutierrez-Merino C. (2014). The Decrease of NAD(P)H:Quinone Oxidoreductase 1 Activity and Increase of ROS Production by NADPH Oxidases Are Early Biomarkers in Doxorubicin Cardiotoxicity. Biomarkers.

[159] Busso I.T., Silva G.B., Carreras H.A. (2016). Organic Compounds Present in Airborne Particles Stimulate Superoxide Production and DNA Fragmentation: Role of NOX and Xanthine Oxidase in Animal Tissues. Environ. Sci. Pollut. Res..

[160] Kim C., Ashrap P., Watkins D.J., Mukherjee B., Rosario-Pabón Z.Y., Vélez-Vega C.M., Alshawabkeh A.N., Cordero J.F., Meeker J.D. (2022). Maternal Metals/Metalloid Blood Levels Are Associated with Lipidomic Profiles Among Pregnant Women in Puerto Rico. Front. Public Health.

[161] Corsetto P.A., Ferrara G., Buratta S., Urbanelli L., Montorfano G., Gambelunghe A., Chiaradia E., Magini A., Roderi P., Colombo I. (2016). Changes in Lipid Composition during Manganese-Induced Apoptosis in PC12 Cells. Neurochem. Res..

[162] Salzer U., Prohaska R. (2001). Stomatin, Flotillin-1, and Flotillin-2 Are Major Integral Proteins of Erythrocyte Lipid Rafts. Blood.

[163] Kwiatkowska K., Matveichuk O.V., Fronk J., Ciesielska A. (2020). Flotillins: At the Intersection of Protein S-Palmitoylation and Lipid-Mediated Signaling. Int. J. Mol. Sci..

[164] Eum S.Y., Jaraki D., András I.E., Toborek M. (2015). Lipid Rafts Regulate PCB153-Induced Disruption of Occludin and Brain Endothelial Barrier Function through Protein Phosphatase 2A and Matrix Metalloproteinase-2. Toxicol. Appl. Pharmacol..

[165] Ni I., Ji C., Vij N. (2015). Second-Hand Cigarette Smoke Impairs Bacterial Phagocytosis in Macrophages by Modulating CFTR Dependent Lipid-Rafts. PLoS ONE.

[166] Winter P.W., Al-Qatati A., Wolf-Ringwall A.L., Schoeberl S., Chatterjee P.B., Barisas B.G., Roess D.A., Crans D.C. (2012). The Anti-Diabetic Bis(Maltolato)Oxovanadium(Iv) Decreases Lipid Order While Increasing Insulin Receptor Localization in Membrane Microdomains. Dalt. Trans..

[167] Park J.-W., Kim H.P., Lee S.-J., Wang X., Wang Y., Ifedigbo E., Watkins S.C., Ohba M., Ryter S.W., Vyas Y.M. (2008). Protein Kinase Cα and ζ Differentially Regulate Death-Inducing Signaling Complex Formation in Cigarette Smoke Extract-Induced Apoptosis. J. Immunol..

[168] Tappe M., Null V. Requirements for Tires from the Environmental View Point. Proceedings of the Tire Technology Expo Conference.

[169] Beretta E., Gualtieri M., Botto L., Palestini P., Miserocchi G., Camatini M. (2007). Organic Extract of Tire Debris Causes Localized Damage in the Plasma Membrane of Human Lung Epithelial Cells. Toxicol. Lett..

[170] Singh D.P., Kaur G., Bagam P., Pinkston R., Batra S. (2018). Membrane Microdomains Regulate NLRP10- and NLRP12-Dependent Signalling in A549 Cells Challenged with Cigarette Smoke Extract. Arch. Toxicol..

[171] Fedida-Metula S., Feldman B., Koshelev V., Levin-Gromiko U., Voronov E., Fishman D. (2012). Lipid Rafts Couple Store-Operated Ca^2+^ Entry to Constitutive Activation of PKB/Akt in a Ca^2+^/Calmodulin-, Src- and PP2A-Mediated Pathway and Promote Melanoma Tumor Growth. Carcinogenesis.

[172] Janes P.W., Ley S.C., Magee A.I. (1999). Aggregation of Lipid Rafts Accompanies Signaling via the T Cell Antigen Receptor. J. Cell Biol..

[173] Ghare S., Patil M., Hote P., Suttles J., McClain C., Barve S., Joshi-Barve S. (2011). Ethanol Inhibits Lipid Raft-Mediated TCR Signaling and IL-2 Expression: Potential Mechanism of Alcohol-Induced Immune Suppression. Alcohol. Clin. Exp. Res..

[174] Goddard A.D., Watts A. (2012). Regulation of G Protein-Coupled Receptors by Palmitoylation and Cholesterol. BMC Biol..

[175] Zheng H., Pearsall E.A., Hurst D.P., Zhang Y., Chu J., Zhou Y., Reggio P.H., Loh H.H., Law P.Y. (2012). Palmitoylation and Membrane Cholesterol Stabilize μ-Opioid Receptor Homodimerization and G Protein Coupling. BMC Cell Biol..

[176] Le Ferrec E., Øvrevik J. (2018). G-Protein Coupled Receptors (GPCR) and Environmental Exposure. Consequences for Cell Metabolism Using the β-Adrenoceptors as Example. Curr. Opin. Toxicol..

[177] Shahid A., Chen M., Lin C., Andresen B.T., Parsa C., Orlando R., Huang Y. (2023). The β-Blocker Carvedilol Prevents Benzo(a)Pyrene-Induced Lung Toxicity, Inflammation and Carcinogenesis. Cancers.

